# Surface Characterisation of Human Serum Albumin Layers on Activated Ti6Al4V

**DOI:** 10.3390/ma14237416

**Published:** 2021-12-03

**Authors:** Margarita Hierro-Oliva, Amparo M. Gallardo-Moreno, María Luisa González-Martín

**Affiliations:** 1 Networking Research Center on Bioengineering, Biomaterials and Nanomedicine (CIBER-BBN), 06006 Badajoz, Spain; margahierro@unex.es (M.H.-O.); mlglez@unex.es (M.L.G.-M.); 2 Department of Applied Physics, Faculty of Science, University of Extremadura, 06006 Badajoz, Spain; 3 University Institute of Extremadura Sanity Research (INUBE), 06006 Badajoz, Spain

**Keywords:** implant material, surface characterization, contact angle, ToF-SIMS, zeta potential, protein adsorption

## Abstract

Adpsortion of protein layers on biomaterials plays an important role in the interactions between implants and the bio-environment. In this context, human serum albumin (HSA) layers have been deposited on modified Ti6Al4V surfaces at different ultraviolet (UV-C) irradiation times to observe possible changes in the adsorbed protein layer. Protein adsorption was done from solutions at concentraions lower than the serum protein concentration, to follow the surface modifications at the beginning of the albumin adhesion process. For this purpose, the surface of the protein-coated samples has been characterized by time of flight secondary ion mass spectrometry (ToF-SIMS), contact angle and zeta potential measurements. The results obtained show a reduction in the total surface tension and zeta potential of samples treated with UV-C light when coated with a protein layer. Furthermore, the UV-C light treatment applied to titanium alloy surfaces is able to modify the conformation, orientation and packing of the proteins arranged in the adsorbed layer. Low irradiation time generates an unstable surface with the lowest protein adsorption and the highest hydrophobic/hydrophilic protein ratio, indicating a possible denaturalization of the protein on these surfaces. However, surface changes are stabilized after 15 h or UV-C irradiation, favoring the protein adsorption through electrical interactions.

## 1. Introduction

Protein adsorption on the implant surface is one of the first biological events that occurs when a foreign material is placed in the human body and comes into contact with body fluids [1,2,3,4]. The adsorption of proteins at biomaterials surfaces is a complex process [5,6,7,8]. Proteins can be attached to a surface in diverse quantities, densities, conformations and orientations. This depends, among other things, on the chemical and physical properties of the material [9]. Some of the most relevant properties of the surface of the material are the composition [10,11], hydrophobicity [10,12,13] and electric potential of interaction known as zeta potential [14,15,16]. For example, studies by some research groups show that proteins tend to adsorb preferentially on hydrophobic surfaces compared to hydrophilic ones [12,17,18]. In particular, human serum albumin (HSA) complies with this behavior according to studies conducted by M. Henry et al. [19]. In addition, the disposition of protein on the surface of the material is also related to the wettability of the surface. A. Hasan et al. in their studies on bovine serum albumin (BSA) ensure that the protein adopts the compact form predominantly on hydrophilic surfaces and the elongated form on the hydrophobic surfaces [20]. On the other hand, the surface charge also affects protein adsorption as it can produce protein attraction or repulsion [21]. Since albumin has a non-homogeneous surface charge distribution its adsorption is possible on both positively and negatively charged surfaces [22].

The adsorbed protein layer plays an important role in the interactions between implants and the bio-environment, and it is directly related to the biocompatibility of the implanted devices. The amount, orientation and conformation of the adsorbed protein layer are crucial for the successful integration of the implant into the body and drive the subsequent interaction of the material with cells [3,23,24]. One of the proteins most widely present in human plasma is albumin, with dimensions of approximately 80 Å × 80 Å × 30 Å in the shape of a heart under physiological conditions and a molecular weight of 66.5 kDa [25,26]. HSA plays an important role in the transport of fatty acids, hormones, metabolites and multiple drugs to various parts of the human body and in the maintenance of osmotic pressure [27,28]. The isoelectric point of this protein was located at pH 5.3 [22]. At physiological pH, albumin has a high net negative charge because the number of acidic amino acid residues exceeds the number the basics ones. The negative charge of the molecule is mainly due to the glutamic and aspartic acid residues, while the positive charge is due to cysteine, tyrosine, lysine and arginine amino acid residues [26,29].

The formation of an adsorbed HSA layer on the surface of some implants can be very useful because the presence of this protein layer reduces the adhesion of bacteria to the implant surface [30,31]. In addition, the presence of an adsorbed HSA layer on the surface of an implant modifies the surface charge of the implant, and this can lead to reduced bacterial adhesion in the case of increasing the to coulomb repulsion between the negatively charged bacteria and the implant surface.

Titanium and some of its alloys have excellent properties as metallic biomaterials [32,33,34]; in particular, they are one of the first choices for the manufacture of dental implants, as they fulfil all biological, chemical and mechanical requirements [35,36]. It is known that the hydrophobicity of the Ti6Al4V biomaterial can be changed after irradiation with ultraviolet (UV-C) light [37,38,39]. This property is related to the presence of titanium dioxide in the passivation layer of the titanium alloy [34,40,41]. TiO_2_ is a semiconductor and it can be excited by ultraviolet radiation of 400 nm or less [42,43]. One of the advantages of UV-C radiation is that it is able to modify the wettability of titanium surfaces without producing changes in the composition, topography or thickness of the passivated layer [44]. In particular, the contact angle of a water drop on the titanium surface decreases to almost zero after 2 h of UV-C irradiation and bactericidal effects can be observed on the Ti6Al4V surface after being subjected to UV-C light during 15 h [44,45]. In addition, the bactericidal effect exhibited by the surface of the Ti6Al4V biomaterial after being subjected to UV-C light during 15 h has been studied [45]. This modification is also accompanied by changes in their surface electrical properties. In this sense, a recent study of our group has shown the huge electronic activity inside the Ti6Al4V passivation layer after irradiation, using electrokinetic techniques based on streaming potential and streaming current. The relative conductivity of the excited TiO_2_ layer versus Ti6Al4V showed a difference of up to five orders of magnitude between the two samples, which could affect any biological adhesion process taking place on its surface [46].

The physical changes on the titanium alloy surface after UV-C irradiation can cause significant changes in the conformation of the protein precursor films to be deposited on the biomaterial in an in vitro situation, and this can condition its increased/decreased biocompatibility. In this sense, time of flight secondary ion mass spectroscopy (ToF-SIMS) technique is a very powerful method to characterize protein layers adsorbed on biomaterial surfaces. Due to its chemical specificity and surface sensitivity, this technique allows the detection of very low amounts of proteins [11,47,48]. In fact, it is possible to use ToF-SIMS to detect different proteins adsorbed on biomaterials based on differences in their amino acids composition [49,50].

The aim of this work is to study the effect of the adsorption of low concentrations of HSA on the surface properties of Ti6Al4V activated by UV-C. To this purpose, ToF-SIMS was used to characterize the adsorption of human serum albumin on two different titanium alloy (Ti6Al4V) surfaces: a native one (TiAlV) and a UV-C treated one (TiAlV UV). The latter was subjected to two different irradiation times, 2 h (TiAlV UV 2h) and 15 h (TiAlV UV 15h). The changes will be related to the modifications of the UV-irradiated surface, mainly in terms of hydrophobicity and zeta potential.

## 2. Materials and Methods

The experiments were carried out with commercially available Ti6Al4V (DKSH, Zürich, Switzerland). A bar of the titanium alloy was cut into discs of 25 mm in diameter and 2 mm in thickness, mechanically polished to mirror finish and carefully cleaned before used. To obtain a mirror finish, the discs were successively abraded using silicon carbide papers and polished with diamond paste, finishing with colloidal silica (all polishing materials were supplied by Buehler, Leinfelden-Echterdingen (Esslingen), Germany). After they were polished, the samples were ultrasonically cleaned in successive baths of distilled water, acetone (Panreac, Castellar del Vallès (Barcelona), Spain) and ethanol (Panreac, Castellar del Vallès (Barcelona), Spain) to remove contaminants from the metal surface. Some titanium alloy surfaces were treated with an ultraviolet lamp for 2 and 15 h in order to make them hydrophilic. A total of 72 samples were analysed, of which one third (24 samples) were not treated with UV-C light (TiAlV), another third were irradiated for 2 h (TiAlV UV 2h) and the last third for 15 h (TiAlV UV 15h) to carry out each experiment in triplicate.

For each of these three groups of samples, adsorbed protein layers of different concentrations were deposited on the samples surfaces. Protein adsorption experiments were performed at 37 °C for 2 h of incubation, in phosphate buffered saline (PBS) onto titanium alloy substrates, using 1 mL of solution with a concentration of 10, 30, 80 and 100 µg/mL of HSA (Sigma-Aldrich, Merck, Darmstadt, Germany) for ToF-SIMS measurements (analysing a total of 36 samples) and 30 µg/mL for zeta potential and contact angle measurements (analysing a total of 18 samples for each technique, including the control samples without adsorbed protein layer). After adsorption, the samples were rinsed three times in deionized water to remove loosely bound protein and buffer salts and finally dried in air.

ToF-SIMS measurements were performed with a ToF-SIMS^5^ (ION TOF, Münster, Germany) equipped with a Bi^+^ primary ion source operating at 25 kV. Four positive ion spectra were taken from each sample surface and three different samples were taken for each condition (in total 12 positive ion spectra of each condition). The total ion dose used to acquire each spectrum was ~1 × 10^12^ ions/cm^2^ to ensure static SIMS conditions with an analyzed area of 150 × 150 μm^2^. The spectra were calibrated for H^+^, H_2_^+^, CH_3_^+^, C_2_H_3_^+^, C_3_H_5_^+^ and C_7_H_7_^+^ peaks before further analysis.

The contact angles on discs were measured using the sessile drop technique with a Drop Shape Analyzer-DSA100E system (Krüss, Hamburg, Germany). ToF-SIMS and contact angles measurements were conducted at the ICTS “NANBIOSIS” (Surface Characterization and Calorimetry Unit) of the CIBER-BBN, at the University of Extremadura. The liquids deionized water (W, Sigma-Aldrich, Merck, Darmstadt, Germany) and diiodomethane (D, Sigma-Aldrich, Merck, Darmstadt, Germany, Germany) were used to measure the static contact angles at room temperature. The surface tension and components of these liquids were published by various authors [51,52]. These values, together with the measured contact angles of the two liquids, were used to calculate the surface tension and components of the samples using the Owens-Wendt-Kaelble (OWK) approach together with Young’s equation [53,54]:

(1) $$\gamma_{L}\left(1+cos\theta \right)=2\sqrt{\gamma_{S}^{d}\gamma_{L}^{d}}+2\sqrt{\gamma_{S}^{nd}\gamma_{L}^{nd}}$$

where $\gamma_{L}$ is the liquid surface tension; *θ* the contact angle of the liquid-solid-air system; $\gamma^{d}$ the dispersive components of the solid and liquid (indicated by the subscripts *S* and *L*, respectively) and $\gamma^{nd}$ the non-dispersive components of the solid and liquid (indicated by the subscripts *S* and *L*, respectively). For the OWK approach, the dispersive component includes the contribution of the London force and the non-dispersive component the contributions of the Keesom, Debye and hydrogen bonding forces, where the total surface tension is the sum of the dispersive and non-dispersive components.

The degree of hydrophobicity of a given material can be expressed as the free energy of interaction between two surfaces of the same material when immersed in water (∆*G_W_*):

(2) $$∆G_{W}=\gamma_{S}-\gamma_{SL}-\gamma_{L}\;,\;\gamma_{SL}=\gamma_{S}+\gamma_{L}-2\sqrt{\gamma_{S}^{d}\gamma_{L}^{d}}-2\sqrt{\gamma_{S}^{nd}\gamma_{L}^{nd}}$$

Taking this parameter into account, a surface is considered hydrophobic when ∆*G_W_* < 0 mJ/m^2^ and hydrophilic when ∆*G_W_* > 0 mJ/m^2^ [55].

Previous studies showed that, after applying UV-C light to Ti6Al4V surfaces for 2 and 15 h, the water contact angle decreases to approximately zero and the surfaces become superhydrophilic [44]. In this study, the contact angle values of the control samples (TiAlV, non-treated and treated with UV-C light) were not measured immediately after irradiation. Thus, the contact angle values were measured after the time required for the protein adsorption experiment had elapsed to check whether the change produced was permanent.

Zeta potential measurements were carried out on an Electrokinetic Analyzer (Anton Paar, Graz, Austria) in terms of streaming current, using a symmetric cell. As previously stated, we measured the streaming current to compare the zeta potential of the metallic samples from the following expression [56]:

(3) $$\zeta =\frac{\eta }{\varepsilon_{r}\varepsilon_{0}}\times \frac{L}{S}\times \frac{dI_{str}}{dp}$$

*ε_r_*, *ε*_0_ and *η* being relative permittivity of the electrolyte solution, vacuum electric permittivity and solution viscosity. *dI_str_*/*dp* was the slope of the streaming current (*I_str_*) vs. pressure (*p*) plot. The electrogeometrical factor, *L*/*S*, is taken as constant for comparisons between samples [57]. Pressure was applied in a differential form (ramp form) from 0 to 600 mbar. Measurements of zeta potential were carried out before and after the protein coating (30 μg/mL) and the electrolyte selected was the standard one KCl 10^−3^ M. All the experiments were carried out by triplicate using three sets of two uncoated and coated disks.

## 3. Results

### 3.1. Protein Adsorption and Structural Reorganisation

Time of flight secondary ion mass spectrometry can be used to characterise adsorbed protein films due to its high chemical selectivity and surface sensitivity. However, the difficulty in interpreting protein mass spectra is their complexity. This is because of the structure of the protein: it consists of a combination of twenty amino acids, and these can dissociate into numerous fragments under ToF-SIMS operation. Hence, the intensities of many different mass to charge ratios (*m*/*z*) have to be taken into account for the analysis. One way to reduce the number of variables is to focus on the most intense peaks of each fragment corresponding to a selected individual amino acid. This information is shown in the Table 1, for HSA-coated TiAlV, where the most intense characteristic peaks are listed, along with their *m*/*z* ratio. This information is also included for the Ti^+^ ion.

**Figure 1:**
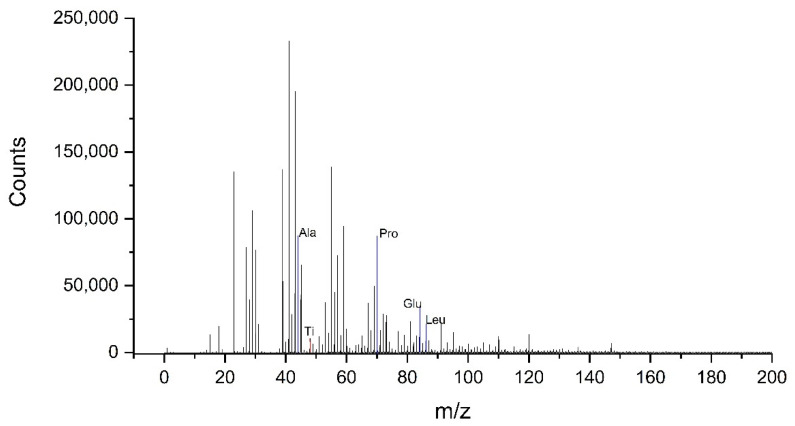
Positive ion time of flight secondary ion mass spectrometry (ToF-SIMS) spectra of the TiAlV surface coated with a solution with 10 µg/mL of HSA concentration.

Figure 1 shows a mass spectrum of cations acquired on a TiAlV sample coated with a solution containing 10 µg/mL concentration of HSA. The presence of HSA is observable in the spectrum through the intensity of the characteristic peaks related to different amino acids present in the protein, such as Alanine, Proline or Leucine.

The peaks appearing in the mass spectra of the hydrophilic sample treated with the same protein layer are very similar to those of untreated one. The rest of the protein concentrations studied also present a spectrum very similar to this one, only differences in the intensities of the peaks are observed.

**Figure 2:**
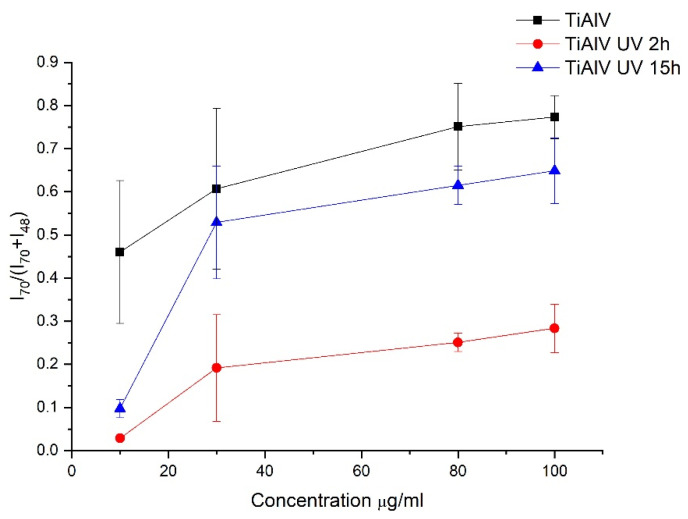
Comparison of the intensity ratio between the *m*/*z* = 70 peak and the sum of the *m*/*z* = 70 and the *m*/*z* = 48 peak as a function of HSA concentration for the native surface (TiAlV) and the two hydrophilic surfaces (TiAlV UV 2h and TiAlV UV 15h).

To evaluate the adsorption of proteins on each titanium alloy surface subjected to different irradiation times, the evolution of the intensity ratio of a characteristic protein peak (Pro, C_4_H_8_N^+^, *m*/*z* = 70) over the sum of a characteristic substrate peak (Ti^+^, *m*/*z* = 48) and the protein peak (*m*/*z* = 70) was followed as a function of HSA concentration for the different samples (Figure 2).

In general, for TiAlV surfaces (non-subjected and subjected to ultraviolet irradiation), the curves of Figure 2 show an increasing tendency of protein adsorption with respect to the concentration of the solution, which is a normal behavior in these adsorption processes [10]. Regarding the UV-C light treatment to which the surfaces were subjected, it could be observed that there is greater protein adsorption on the native surfaces (TiAlV) than on the UV-C treated samples. In addition, the sample treated during 2 h with UV-C light shows the lowest affinity for the protein. In the case of the other two surfaces, the native one and the UV-C treated for 15 h, the amount of protein adsorbed for each surface seems to be the same from 30 µg/mL considering the experimental uncertainty obtained. The difference found in the adsorption behaviour on the surfaces irradiated for 2 and 15 h is significantly different, even though both treatments cause surface hydrophilisation. The most significant changes are observed at the lowest concentration (10 µg/mL), for which there is a large difference between the surfaces treated with UV-C light and the native one. The amount of protein adsorbed on the surface without UV-C treatment at a concentration of 10 µg/mL is five times higher than for treated surfaces. More specifically, there is a decrease in the amount of adsorbed protein, compared to the control sample (TiAlV), of 93% for the surface irradiated during 2 h (TiAlV UV 2h) and 78% for the surface irradiated during 15 h (TiAlV UV 15h). At the concentration of 30 µg/mL, the amount of adsorbed protein decreases by 69% for the TiAlV UV 2h sample and by 13% for the TiAlV UV 15h sample. These percentages of reduction in the adsorbed protein amount are maintained for concentrations of 80 µg/mL and 100 µg/mL. Therefore, changes in the amount of adsorbed proteins due to sample treatment are much more constant and stable from 30 µg/mL. For this reason, 30 µg/mL of concentration is chosen for the contact angles and zeta potential measurements.

**Figure 3:**
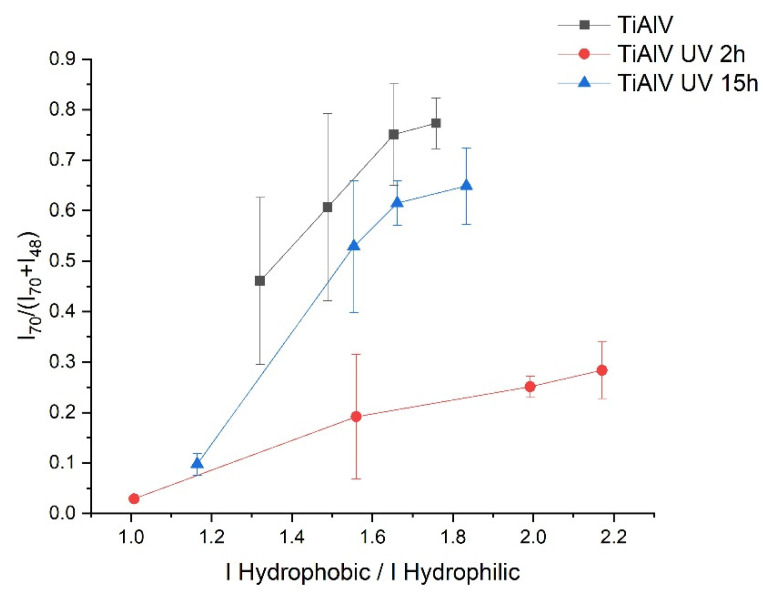
Ratio between peak intensities of amino acids with hydrophobic side chains (Met, Pro, Leu) and amino acids with hydrophilic side chains (His, Thr, Tyr) as a function of the amount of protein adsorbed (I_70_/(I_70_+I_48_)).

In order to detect conformational changes, the ratio of peak intensities is calculated by the sum of amino acids with hydrophobic side chains (Met, Pro, Leu) to the sum of amino acids with hydrophilic side chains (His, Thr, Tyr) [58,59]. The result of this calculation versus the amount of protein adsorbed can be seen in Figure 3.

The ratio hydrophobic/hydrophilic side chains seems to increase with protein concentration in any case, but it is the highest for the surface irradiated 2 h, which means that by increasing the protein concentration the hydrophobic groups of the protein are more exposed to the surface than the hydrophilic ones.

In the case of the other surfaces (TiAlV and TiAlV UV 15h), the ratio between hydrophobic and hydrophilic groups reaches lower values for higher protein concentrations, and both surfaces behaves in a more similar.

### 3.2. Surface Hydrophobicity

In order to characterise the surfaces in terms of hydrophobicity, Table 2 shows the contact angle of deionized water (*θ_W_*) and diiodomethane (*θ_D_*) on TiAlV samples, TiAlV after HSA adsorption and TiAlV after UV-C treatment without and with protein adsorption. Equations (1) and (2) were used to calculate the surface tension components, the total surface tension ($\gamma_{S}$) and the free energy of interaction (∆*G_W_*), also shown in Table 2. The surfaces that have not been subjected to UV-C treatment (TiAlV and TiAlV + HSA) have values for water contact angles sufficiently high to indicate a hydrophobic character. Bearing in mind the experimental uncertainty, they seem to have a tendency to slightly decrease their average water contact angle with the presence of the protein surface layer (TiAlV + HSA). The average surface tension for TiAlV + HSA seems to increase in relation to TiAlV and this is mainly due to the increase in the non-dispersive component ($\gamma^{nd}$). The hydrophobic character of these samples is verified by the calculation of the free energy of interaction as it is minor to zero.

In general, most characterisation studies performed on treated surfaces that will subsequently come into contact with proteins or other organic molecules are performed immediately after surface modification. Nevertheless, to fully complete the adsorption experiments, the surface and the solution must be in contact for a period of time of several hours. The time taken to complete this experiment after surface modification may be important for surfaces that are unstable or whose modification does not cause permanent effects, as in the case of UV-C light irradiation on titanium samples [60,61]. For this reason, samples that were not in contact with proteins were measured after the time of the protein adsorption experiment had elapsed in order to check whether the effect of the surface treatment remained after that time. In all this, it appears that samples subjected to UV-C treatment undergo significant changes in their hydrophobic behavior (*θ_W_* in Table 2). After 2 h of UV-C irradiation, compared to the non-irradiated sample (TiAlV), *θ_W_* changes from 74 ± 5° to 46 ± 5° and *θ_D_* also decrease, in average, 23°. It is also interesting to highlight the increase in the $\gamma^{nd}$ component (2.3 times higher than for non-irradiated sample). In addition, more moderate increases are observed in the dispersive component,$\;\gamma^{d}$, which is 1.4 times higher than for non-irradiated sample. All these changes cause the total surface tension, $\gamma_{S}$, to increase from a value of 40 mJ/m^2^ for the control sample (TiAlV) to 62 mJ/m^2^ for the sample irradiated for 2 h (TiAlV UV 2h). On the other hand, the TiAlV UV 15h sample remains superhydrophilic after the time of the adsorption experiment and shows a water contact angle close to zero even though it was measured 2 h after irradiation finished. A considerable increase in the dispersive and non-dispersive components of the sample TiAlV UV 15h is also observed. In this case,$\;\gamma^{nd}$ is 4 times higher than for the non-irradiated sample, which is correlated with the gradual coating of the surface with hydroxyl groups, causing the surface to acquire a hydrophilic character. Thus, the, $\gamma_{S}$ of this sample is much higher than for the previous ones, reaching a value of 79 mJ/m^2^ (Table 2), by consequence, ∆*G_W_* for this sample becomes zero/positive indicating a hydrophilic like behavior.

Considering the results obtained for the samples irradiated in contact with HSA, significant changes in the water contact angle are also observed. For these samples, starting from a hydrophilic surface after UV-C treatment, as discussed in the preceding paragraph, the water contact angle increases for the same samples with the adsorbed protein layer. This change is remarkable for the TiAlV UV 15h sample, as it moves from a superhydrophilic surface (*θ_W_* ≈ 0°) to a water contact angle of 72 ± 2° for the protein-adsorbed layer (Table 2). Further noticeable variations are also observed for *θ_D_*, increasing 17° and 24° for samples irradiated with UV-C for 2 h and 15 h, respectively, when the adsorbed protein layer is present. These changes are reflected in the components of surface tension. The dispersive component decreases considerably when there is a protein layer on the surfaces treated with UV-C light. On the other hand, the changes for the non-dispersive component are very significant for the TiAlV UV 15h sample; this component is 4 times smaller when there is a protein coating. This translates into a huge change for the surface tension of the solid, being 1.8 times lower for the sample irradiated for 15 h with the adsorbed protein layer than for the surface without protein.

### 3.3. Electric Potential of Interaction

**Figure 4:**
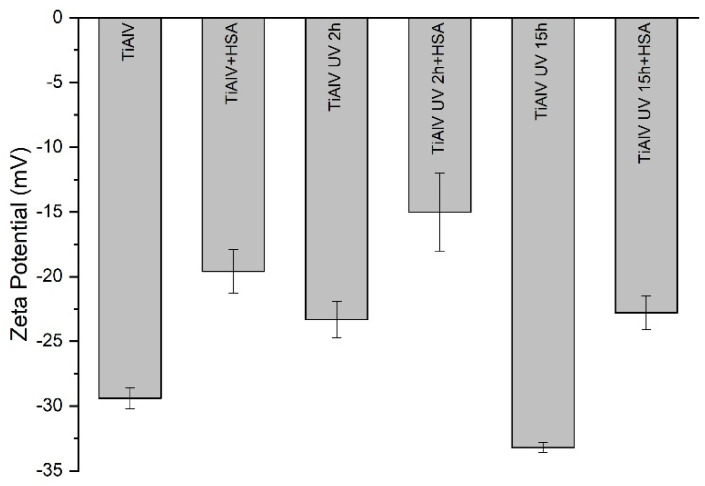
Zeta potential values for different surfaces, with different treatments, with and without adsorbed protein layer.

Figure 4 shows the zeta potential of uncoated and protein coated TiAlV surfaces without and with irradiation. In all cases, the surfaces are negatively charged, and the amount of the absolute charge depends on the surface treatment. Comparing the surfaces without protein coating, 2 and 15 h of UV-C irradiation cause a different effect on the surface charge of TiAlV: 2 h of irradiation decreases the negative surface charge from −29.4 ± 0.8 mV to −23.3 ± 1.4 mV and 15 h of irradiation increases the negative surface charge from −29.4 ± 0.8 mV to −33.2 ± 0.4 mV. The experimental uncertainties for the samples treated with 2 h of UV-C irradiation are higher than for samples treated with 15 h of irradiation, which may be related to the very low stability of surfaces after a short irradiation time. As observed in the contact angle measurements, the surface of the TiAlV UV 2h sample, once the time of the protein adsorption experiment has elapsed, is unstable and returns to its initial state.

After the protein adsorption, the surfaces become less negatively charged in any case. In general, measurements with protein layers have more experimental uncertainty than without proteins and TiAlV UV 2h sample is the case with the highest: ±3 mV. It is also interesting to note that the ζ changes after protein adsorption are similar for the three groups of surfaces: 10 ± 3 mV for TiAlV, 8 ± 4 mV for TiAlV UV 2h, and 10.3 ± 1.6 mV for TiAlV UV 15h, which means that the effect of protein adsorption on all surfaces on the zeta potential is similar, about 10 mV.

## 4. Discussion

The decreasing of water contact angle when there is an adsorbed HSA layer is in line with the observations of M. Müller et al. in their studies carried out on the adsorption of plasma proteins onto cellulosic substrates [62]. It has been described that albumin adsorption is strongly influenced by the surface properties of the material on which adsorption takes place [63]. In particular, albumin has been found to have more affinity for hydrophobic than for hydrophilic substrates, attributing these changes to the significant dehydration of hydrophobic surfaces as protein adsorbs [18]. In line with these researchers, if the irradiated surfaces are hydrophilised in relation to the non-irradiated surface, a decrease in the amount of proteins adsorbed to both surfaces could be predicted, especially in the case of TiAlV UV 15h sample. However, there is only a decrease in the adsorbed protein quantity on the TiAlV UV 2h sample (Figure 2) and the amount of proteins adsorbed after 15 h of irradiation is similar to the control. This difference in the amount of protein adsorbed on the respective surfaces might be the result of a structural reorganisation of the HSA molecules due to the UV-C light treatment, which could lead to changes in the conformation, orientation and packing of the proteins arranged in the layer. The different hydrophobic/hydrophilic ratios observed in Figure 3 can point out that the protein will have different orientations depending on the treatment applied to the surface. In particular, the hydrophobic groups of the protein adsorbed on the surface irradiated for 2 h are more exposed on the surface than the hydrophilic ones. According to Tidweel et al. [64], for proteins in solution or simulating a biological environment, the amino acids with hydrophobic side chain will be more concentrated in the core of the protein and the amino acid with hydrophilic side chain will be more concentrated in the outer regions of the protein. Therefore, changes in the conformation of the protein, such as denaturation, will result in changes in the ratio between the intensities of the hydrophobic and hydrophilic groups of the protein. Based on this statement and taking into accounts the results of Figure 3, it appears that HSA can be denatured when in contact with the 2 h irradiated surface, and this can be related to the lowest amount of adsorbed protein in this case. However, in the case of HSA adsorbed on the surface after 15 h of UV-C radiation, a greater exposure of hydrophobic groups, as it would be expected due to the superhydrophilicity of TiAlV UV 15h, than in albumin adsorbed on the non irradiated sample is not observed.

Previous studies of our group on Ti6Al4V proved that excitation by UV-C irradiation for a long period of time (15 h) modifies the properties of its passivation layer [44]. It was found that the Ti6Al4V surface after irradiation, besides reaching a superhydrophilic state, as it happens after short irradiation times (2 h), also shows bactericidal properties [45]. In that work we also found that these modifications in the surface properties are related to the semiconductor nature of the passivation layer present on the surface of titanium alloy. This passive layer, as pure TiO_2_, is susceptible to modify its electronic distribution in the valence and the conductive bands upon excitation with UV-C irradiation. The modification of the electronic distribution in the passive layer after irradiation for 15 h results in high electronic activity on the surface of the Ti6Al4V sample [46]. The conductivity of the passive layer of the Ti6Al4V surfaces changes from values of dielectric nature, before irradiation with UV-C light, to those of conductive materials. Therefore, it can be expected that protein adsorption on the excited surface of Ti6Al4V is driven by both hydrophobic/hydrophilic and electrical interactions acting together in the adsorption process. The fact that adsorption on the superhydrophilic surface (irradiated 15 h) is similar to adsorption on the untreated samples indicates that the extra electrical contribution of this surface is able to surmount the adsorption inhibition effect that is expected on hydrophilic surfaces, as is the case for the surface irradiated 2 h. Therefore, despite this strong and profound modification of the passive Ti6Al4V layer produced by a period of long-term UV-C irradiation, once this UV-C irradiation ceases, the system gradually returns to its original electronic configuration, as the excited electronic state of the passive layer is relaxed. This electronic decay is associated with an emission of radiation at a wavelength longer than or equal to the excitation UV-C wavelength. The effect of this decay radiation on the damage of bacterial viability has been assessed [45]. As well as for the survival of bacteria, the UV-C radiation affects proteins. It has been demonstrated that UV-C radiation on albumin solutions to cause conformational rearrangement and aggregation of the protein’s polypeptide chains [65]. From this perspective, it can be suggested that the possible radiative emission associated with electronic decay in the passivation layer may be promoting aggregation of the chains in the protein or at least preventing the elongation of the protein associated with the hydrophilicity of the substrate to which it is adsorbed.

From the results obtained after studying the hydrophobicity of the samples, it is remarkable that TiAlV UV 2h does not become superhydrophilic, as obtained in other studies carried out by the research group [44], in which the contact angle of water on Ti6Al4V samples irradiated for 2 h with UV-C light is almost zero. This indicates that the surface of the TiAlV UV 2h sample, once the time of the protein adsorption experiment has elapsed, is unstable and returns gradually to the initial values of the contact angle. This may be related to the fact that the protein is denatured on this surface, as can be deduced from Figure 3, in which states that the hydrophobic groups of the protein adsorbed on the sample irradiated for 2 h are more exposed on the surface than the hydrophilic ones.

Considering the results of the electrical surface characterization based on the zeta potential, it appears that the proteins are “neutralising” a similar number of negative charges on the surfaces, regardless of the amount of irradiation previously received from the surface. Bearing in mind that the amount of proteins adsorbed in TiAlV UV 2h is lower than in the other cases, the protein molecules must be conformed on the surface in a different way than in the other two cases (TiAlV and TiAlV UV 15h). This can be also in line with the possible denaturalization of the protein: a more distended-elongated configuration of the proteins is proposed in the case of 2 h of irradiation compared to the configuration adopted on the surfaces irradiated for 15 h and non-irradiated. The different electrical behavior of the surfaces subjected to different irradiation times (2 and 15 h) can also be related to the different behavior of both surfaces against bacteria: only in the case of irradiation with UV-C light for 15 h, the surfaces exhibit bactericidal behavior against different bacteria strains [66].

Adsorption of protein layers on activated Ti6Al4V surfaces by means of UV irradiation can be different depending on the time of irradiation. Precursor protein layers on instable excited surfaces can lead to protein denaturalization, which could alter the next adhesive processes with other biological materials such as cells.

## 5. Conclusions

The amount of UV-C irradiation to which the samples are subjected is decisive in the changes observed in the adsorbed protein layer. UV irradiation of TiAlV surfaces for a long period of time (15 h) allows activating the passivation layer, achieving surfaces that, together with an interesting bactericidal potential, do not compromise the adsorption of albumin, neither in quantity nor in configuration.

Protein-coated surfaces are always less negatively charged, no matter the surface irradiation-dose. Moreover, as the charge balance observed on the irradiated surfaces is always the same, a more distended-elongated configuration of the proteins is proposed in the case of 2 h of irradiation compared to the configuration adopted on the surfaces irradiated for 15 h.

## Figures and Tables

**Table 1:** Most intense peaks characteristic of the proteins, observed in all positive spectra of the titanium alloy coated with human serum albumin (HSA) protein.

Ion Fragments	*m*/*z*	Ion Fragments	*m*/*z*
CH_4_N^+^ (Glycine, Gly)	30.036	C_4_H_5_N_2_^+^ (Histidine, His)	81.048
C_2_H_6_N^+^ (Alanine, Ala)	44.052	C_4_H_6_NO^+^ (Glutaic Acid, Glu)	84.051
Ti^+^	47.948	C_5_H_12_N^+^ (Leucine, Leu)	86.107
C_4_H_5_O^+^ (Threonine, Thr)	69.038	C_3_H_7_N_2_O^+^ (Asparagine, Asn)	87.067
C_4_H_8_N^+^ (Proline, Pro)	70.072	C_3_H_6_NO_2_^+^ (Aspartic Acid, Asp)	88.047
C_4_H_10_N^+^ (Valine, Val)	72.087	C_4_H_10_N_3_^+^ (Arginine, Arg)	100.092

**Table 2:** Contact angles values for deionized water (*θ_W_*) and diiodomethane (*θ_D_*) obtained on the surfaces of TiAlV, TiAlV treated with UV-C light and TiAlV with adsorbed protein layer. Also shown in the table are the dispersive $(\gamma^{d}$ ) and non-dispersive ($\gamma^{nd}$ ) components of the surface tension, the total surface tension ($\gamma_{S}$ ) of the solid, calculated using the Owens-Wendt-Kaelble (OWK) approach and the free energy of interaction between two surfaces (∆*G_W_*).

Sample	*θ_W_* (°)	*θ_D_* (°)	$\gamma^{d}\;(\mathrm{mJ}/m^{2})$	$\gamma^{nd}\;(\mathrm{mJ}/m^{2})$	$\gamma_{S}\;(\mathrm{mJ}/m^{2})$	∆*G_W_* (mJ/m^2^)
TiAlV	74 ± 5	54 ± 3	32 ± 2	8 ± 3	40 ± 5	−53 ± 9
TiAlV + HSA	62 ± 8	48 ± 9	35 ± 5	13 ± 7	48 ± 11	−38 ± 17
TiAlV UV 2h	46 ± 5	31 ± 3	44 ± 1	19 ± 3	62 ± 5	−22 ± 6
TiAlV UV 2h + HSA	51 ± 21	48 ± 7	35 ± 4	20 ± 15	55 ± 19	−27 ± 27
TiAlV UV 15h	0	24 ± 2	47 ± 1	33 ± 1	79 ± 1	0 ± 1
TiAlV UV 15h + HSA	72 ± 2	48 ± 4	35 ± 2	8 ± 2	43 ± 4	−50 ± 6

## Data Availability

Data is contained within the article.
